# Disparities in Reproductive Aging and Midlife Health between Black and White women: The Study of Women’s Health Across the Nation (SWAN)

**DOI:** 10.1186/s40695-022-00073-y

**Published:** 2022-02-08

**Authors:** Siobán D. Harlow, Sherri-Ann M. Burnett-Bowie, Gail A. Greendale, Nancy E. Avis, Alexis N. Reeves, Thomas R. Richards, Tené T. Lewis

**Affiliations:** 1 Department of Epidemiology, University of Michigan, School of Public Health, United States, 1415 Washington Heights, Ann Arbor, MI 48104-2029 USA; 2 Endocrine Division, Department of Medicine, Massachusetts General Hospital, Harvard Medical School, Boston, USA; 3 Division of Geriatrics, David Geffen School of Medicine, University of California at Los Angeles, Los Angeles, USA; 4 Department of Social Sciences & Health Policy Wake Forest School of Medicine, Winston-Salem, USA; 5 Department of Epidemiology, School of Public Health, University of Michigan, Ann Arbor, USA; 6 Department of Epidemiology, Rollins School of Public Health, Emory University, Atlanta, USA

**Keywords:** Menopause, Midlife, Racism, Race, Discrimination, Hot flash, Cardiovascular disease, Metabolic disease, Mental health, Sleep

## Abstract

This paper reviews differences in the experience of the menopause transition and midlife health outcomes between Black and White women who participated in the Study of Women’s Health Across the Nation (SWAN), a 25-year, longitudinal, multi-racial/ethnic cohort study. We identify health disparities, i.e., instances in which Black women’s outcomes are less favorable than those of White women, and consider whether structural racism may underlie these disparities. Although SWAN did not explicitly assess structural racism, Black women in SWAN grew up during the Jim Crow era in the United States, during which time racism was legally sanctioned. We consider how we might gain insight into structural racism by examining proxy exposures such as socioeconomic characteristics, reports of everyday discrimination, and a range of life stressors, which likely reflect the longstanding, pervasive and persistent inequities that have roots in systemic racism in the US. Thus, this paper reviews the presence, magnitude, and longitudinal patterns of racial disparities observed in SWAN in six areas of women’s health – menopause symptoms, sleep, mental health, health related quality of life, cardio-metabolic health, and physical function –and elucidates the contextual factors that are likely influencing these disparities. We review the strengths and weaknesses of SWAN’s design and approach to analysis of racial disparities and use this as a springboard to offer recommendations for future cohort studies.

## Introduction

Over the past three decades, scientific understanding of the natural history of menopause and midlife aging has increased dramatically [1–4] with specific attention given to extending knowledge from the experiences of White American women to include the experiences of Black American women and other populations in the United States (US). The menopause transition (MT), defined as the transition from reproductive to non-reproductive status, occurs during midlife, demarcated by the ages of ~ 40 to ~ 60 years. The MT and midlife are critical life-stages for two principal reasons. First, most US women experience vasomotor symptoms (also known as hot flashes and night sweats), and some may experience depressive symptoms, sleep disorders, and anxiety, all of which impact quality of life and, in some instances, increase risk of chronic diseases [5–9]. Second, during midlife, the incidence of chronic diseases, such as osteoarthritis and cardiovascular disease, accelerate. [10, 11]

One of the hallmark US studies of menopause is The Study of Women’s Health Across the Nation (SWAN), a multi-site, multi-racial/ethnic cohort of women who were enrolled in 1996 at the age of 42 to 52 years and followed for over 25 years, as participants transitioned from pre-menopause through menopause and into early old age [1]. This review focuses on understanding health disparities between Black and White SWAN women during the MT and midlife by conducting a synthetic review of SWAN publications. We summarize here the differences observed between Black and White SWAN participants in the occurrence of MT symptoms, selected chronic diseases, physical function, and health-related quality of life (HRQL). Specifically, we aimed to elucidate those symptoms and conditions for which Black women’s symptom experience and health outcomes were less favorable than those of White women to document potential racial midlife health disparities. Although several SWAN publications discuss racial differences in specific outcomes, no prior publication has summarized the range and magnitude of findings regarding potential health disparities across the multiple domains assessed in SWAN. This paper synthesizes SWAN’s findings with respect to Black-White disparities in the age at which menopause occurs and in six domains that are in flux during the MT and mid-life: vasomotor symptoms (VMS), depressive symptoms, sleep characteristics, cardiometabolic health, physical function, and health-related quality of life.

Furthermore, we propose that some of these observed disparities are likely attributable to structural racism in the US. First, we acknowledge explicitly the socio-historical experience of SWAN participants, as most Black women in SWAN were born in the United States and grew up during the Jim Crow era when racism was legally sanctioned. Second, although SWAN did not explicitly assess structural racism, we consider how we can gain indirect insight into structural racism by examining Black-White differences in germane proxy exposures such as socio-economic status, life stressors, and differential access to medical care that likely reflect the longstanding, pervasive and persistent inequities stemming from systemic racism in the US [12, 13].

Quantifying Black-White differences in midlife health trajectories, and the potential factors underlying these differences, is central to promoting optimal aging for all women. Health trajectories remain malleable during the midlife, thus the identification of unfavorable risk factors and the adoption of appropriate prevention and intervention strategies can increase the likelihood of successful aging [14–19]. Finally, we consider the strengths and weaknesses of SWAN’s design and approach to analyzing racial differences and use this reflection as a springboard to offer recommendations for future studies.

### The Socio-Historical Context of SWAN Participants’ Life Experiences.

To understand Black-White differences in SWAN findings, it is essential to consider the social context of SWAN participants’ lives prior to study enrollment. SWAN women were born between 1944 and 1954, and thus grew up in a US society shaped by the structural, or institutional, racism embodied in Jim Crow laws [20, 21].^1^ These laws sanctioned racial discrimination and segregation in housing, education, employment, and health care for Black Americans [20]. Moreover, these laws sanctioned a climate of terror in which Black citizens were subject to lynching and other overt violence [20]. Thus, many of the Black women in SWAN likely grew up in segregated neighborhoods, were educated in segregated schools, and came of age prior to the passing of the landmark 1964 Civil Rights Act that outlawed discrimination in the US based on race [22].

Although the Civil Rights Act of 1964 was a major breakthrough for civil liberties, it is widely acknowledged that substantial disparities in education, economic opportunity, and access to healthcare persisted long after its passage, when Black women in SWAN were adolescents and young adults. Thus, a central hypothesis of this review is that structural racism, or ‘differential access to the goods, services, and opportunities of society by race’, is a major contributor to health disparities in the cohort [13, 23]. SWAN did measure everyday discrimination, but did not formally measure structural racism, i.e., SWAN did not assess differential educational quality, access to jobs, or lack of opportunity to live in specific neighborhoods (e.g., “redlining” and deed covenants). However, SWAN did ascertain potential proxy measures that characterize some of the downstream effects of structural racism in the form of social disadvantage such as lesser educational attainment, lower socioeconomic status (SES), financial strain, etc. Thus, our theoretical model holds that systematic differences in these proxy measures by race/ethnicty group can be used to gauge some of the impact of structural racism on the midlife experience of Black SWAN participants [12, 13]. Notably, most current measures of structural racism are in fact indirect or proxy measures (e.g. percent of people registered to vote, percent of elected officials who are Black/African-American) [24] because the Jim Crow laws under which most SWAN women came of age have been repealed.

## Methods

### Approach to Review

As of March 2021, SWAN investigators had reported over 590 publications (a complete list is available at https://www.swanstudy.org/publications/). First, we identified all publications on this list that included the words race or race/ethnicity, discrimination, or childhood adversity in the title and/or abstract. We next limited the list to those publications that included analyses evaluating racial/ethnic differences in the prevalence, incidence, severity, or timing of outcomes of interest; publications that examined racial/ethnic interactions or conducted race-stratified analyses; and publications that assessed the impact of discrimination or childhood socioeconomic adversity. In addition, to ensure that we had not missed relevant manuscripts, we asked investigators who had provided scientific leadership for the specific domains covered in this review to identify manuscripts that had examined racial/ethnic differences. To identify papers from researchers based on SWAN public use data sets, we reviewed publications listed on the National Archive of Computerized Data on Aging (https://www.icpsr.umich.edu/web/NACDA/series/253) SWAN series website. We also searched OVID Medline with the search terms “Study of Women’s Health Across the Nation” and “Ethnic Groups or Race or Racial or African-American”.

SWAN’s primary goals were to describe the natural history of menopause and the impact of the MT on women’s midlife health, with a focus on distinguishing influences attributable to reproductive aging as opposed to chronological aging. Although SWAN enrolled a multi-racial/ethnic cohort (Black, Chinese, Hispanic, Japanese, and White women), it has not systematically examined racial/ethnic disparities in non-reproductive health outcomes, or the impact of discrimination or the potential role of structural racism on health. Thus, we limited this review to outcomes for which racial/ethnic disparities were evaluated and were potentially due to structural racism. Thus, this review considers age at menopause and six health domains, specifically, vasomotor symptoms (VMS), depressive symptoms, sleep characteristics, cardio-metabolic health, physical function, and health-related quality of life. The extent to which SWAN publications investigated potential disparities varied across these outcomes. Next, for each outcome and to the extent results are available, we summarize evidence for disparities in prevalence and incidence, evidence for associations with proxy measures of structural racism, and evidence relating to differences in risk factors by race.

This paper focuses on disparities in the midlife health of Black participants compared to White participants for several reasons. First, given the relatively large sample of Black women in SWAN recruited from four SWAN sites, SWAN is uniquely well-positioned to speak to Black women’s experiences across a range of geographic contexts. Second, the samples of women from Chinese, Japanese and Hispanic racial/ethnic groups were small and each of these groups were recruited at a single SWAN site. Thus, SWAN is not well-positioned to address differences in midlife health between White women and these other groups. Third, given the multiple health domains investigated in this review, the resulting large volume of information, and the differences in midlife health experiences of Black women compared with other groups represented in SWAN, a review of racial/ethnic disparities in health for the Chinese, Japanese, and Hispanic samples was beyond the scope of this paper.

### SWAN Cohort Recruitment and Enrollment

In 1995–97, SWAN conducted a cross-sectional screening survey to identify women eligible for the cohort study. The study was conducted at seven clinical sites. The cross-sectional study screened 16,065 women aged 40 to 55 years with 3302 women aged 42–52 years subsequently enrolled into the SWAN cohort. Each site screened a community-based sample of Black (Boston, MA; Chicago, IL; Pittsburgh, PA, and southeast Michigan, *n* = 935), Chinese (Oakland, CA, *n* = 250), Japanese (Los Angeles, CA, *n* = 281), Hispanic (Newark, NJ, *n* = 286), and 1550 White (all sites) women [1]. For the reasons enumerated above, this review focuses on results for the Black versus White participants only. To be eligible for the cohort, women had to have an intact uterus and at least one ovary, have menstruated and not used hormone therapy (HT) in the previous three months, and not be pregnant. The study protocol was approved by the Institutional Review Board at each clinical site and informed consent was obtained at each study visit. Since the 1996–97 baseline visit, depending on the site, 15 or 16 follow-up visits have been completed, with an average interval of 18 months between follow-ups. The most recent follow-up visit was in 2015–18 and 74% of surviving women participated. At each visit, women completed interviews and self-administered questionnaires; had height, weight and blood pressure measured; and blood drawn.

### Measures

SWAN assessed a broad range of exposures including sociodemographic characteristics, lifestyle characteristics, perceived stress, major life events and everyday discrimination, as well as a range of reproductive health outcomes such as menopause transition stage and vasomotor symptoms [1]. Race/ethnicity was self-defined. Sociodemographic characteristics included marital status, number of children, level of education attained, employment status, and financial strain. Financial strain was measured by the question “how hard is it to pay for basics (very hard, somewhat hard, or not hard)?” Lifestyle characteristics including smoking status, alcohol use and level of physical activity in four domains (occupation, household and caregiving, sports and exercise, and daily routine) assessed by a 19-item version of the Kaiser Physical Activity Survey [25]. Experience of discrimination was assessed by the 10-item Everyday Discrimination Scale, a measure of interpersonal discrimination [26]. The scale inquires about daily mistreatment first, without labeling these episodes as “racism,” or “discrimination”. After reporting their experiences, respondents are asked to attribute them to one or more of the following reasons (race, ethnicity, gender, age, income level, language, body weight, physical appearance, sexual orientation, other). Depressive symptoms were assessed by the Center for Epidemiologic Studies Depression scale (CES-D) (with the standard score of >  = 16 indicative of significant depressive symptoms [27]. SWAN serially assessed subjective sleep quality with a standardized self-report scale [28]; later, when women were average age 65 years, the study added actigraphy, an objective assessment of sleep quality.

The study also assessed domains of general health that may be impacted by midlife aging such as physical symptoms (e.g., pain), chronic conditions (e.g., hypertension, diabetes mellitus), prescription medication use, physical function (e.g., grip strength, timed stair climb, timed 4-m walk), and health-related quality of life assessed by the RAND 36-item Short Form Survey (SF-36) [29]. SWAN initially included 5 of the 8 SF-36 domains (vitality, bodily pain, social function, role limitations due to physical health, and role limitations due to emotional health) but included all 8 domains beginning at the 6^th^ follow-up visit. Inclusion of the 8 domains permitted computation of the standard summary scores –the physical health component score (PCS), that primarily weights the domains of self-assessed health, physical functioning, bodily pain, and role limitations caused by physical health, and the mental health component score (MCS), that primarily weights the domains of vitality, mental health, role limitations caused by emotional health, and social functioning. Notably, the demarcation between symptoms/conditions influenced by reproductive versus (vs.) chronologic aging had not been clearly established, and one of the goals of the longitudinal component of SWAN was to understand the contributions of each kind of aging to the many outcomes assessed.

In addition to self-report and physical measurements, serum was assayed for reproductive hormones, cardio-metabolic markers, adipokines, and inflammatory markers. Measures of subclinical cardiovascular disease, peripheral neuropathy, and objective sleep were undertaken at selected follow-up visits.

### Ancillary studies

Independently funded ancillary studies provided more comprehensive measures of sleep, subclinical cardiovascular disease (CVD), and mental health for subsets of SWAN participants, and augment our understanding of racial differences in selected domains Table 1.

**Table 1:** Sample sizes of Black and White Women by Study Component of the Study of Women’s Health Across the Nation

Study Component	Black Women	White Women
Cohort	935	1550
*Ancillary Studies*		
SWAN Heart	382	226
SWAN Sleep	139	172
SWAN Mental Health		
Cross-sectional	247	514
Longitudinal	146	277
Childhood Context	506	603

In brief, SWAN Heart, a study of subclinical CVD conducted in Chicago and Pittsburgh, enrolled 382 Black and 226 White women between 2001 and 2003. At enrollment, SWAN Heart participants had no history of coronary heart disease or stroke, were not using diabetes medications or hormone therapy (HT), and were not post-menopausal. One to four years later, 462 women were re-assessed. Measures included aortic pulse wave velocity, carotid plaque, aortic calcification, and coronary artery calcification. SWAN Sleep, conducted in Chicago, southeast Michigan, Pittsburgh, and Oakland, included 139 Black and 172 White women. Measures included subjective sleep quality and polysomnography-assessed indices of sleep duration, continuity/fragmentation, depth, and sleep disordered breathing. SWAN Mental Health, conducted in Chicago, Pittsburgh, and New Jersey, utilized the Structured Clinical Interview for DSM-IV [30] to assess major depression and anxiety and included 247 Black and 514 White women [31]. Pittsburgh collected this information longitudinally across 11 visits in 425 women. The SWAN Childhood Context, conducted in Boston, Chicago, Pittsburgh, and southeast Michigan, interviewed 506 Black and 603 White women as part of the 13^th^ follow-up visit [32]. A 10-item questionnaire ascertained information on participants’ maternal and paternal education and other indicators of socioeconomic status during their childhood and adolescence, including whether their family owned a car or a home, ever received public assistance, or had difficulty paying for food or rent [33].

## Results

### Black-White Differences in Life Context as a Mirror of Social Disadvantage

The prior life context—educational attainment, socioeconomic status, childhood and family characteristics, experience of adverse life events, and health-related behaviors – of Black women and White women differed significantly at SWAN enrollment [34]. Black women were less likely to have a college degree (32.5% vs. 53.3%). Additionally, although one-third of Black women in SWAN were college-educated, Black women overall were more likely than White women to report difficulty paying for basics such as rent and food (46.2% vs. 32.0%) and less likely to be employed (79.1% vs. 85.3%) Table 2.

**Table 2:** Life Context of Black and White women at Cohort Enrollment: Study of Women’s Health Across the Nation, 1996–1997 [34]

Life Context	Black Women (*n* = 916)	White Women (*n* = 1533)
*Socio-economic*		
College Degree	32.5%	53.3%
Financial Strain^a^	46.2%	32.0%
Employed	79.1%	85.3%
Childhood Socioeconomic	40.7%	14.6%
Disadvantage ^b^		
*Family Structure*		
Currently Married	47.3%	71.5%
Have Children	91.0%	75.8%
*Lifestyle*		
Current Smoker	24.1%	16.6%
Passive Smoke Exposure (> = 5 person-hours/week)	39.4%	29.1%

^a^Somewhat or very difficult to pay for basics such as food, shelter or heat

^b^SWAN Childhood Context ancillary study *n* = 506 Black and 603 White women[32]

Differences were also present in other socioeconomic and sociocultural characteristics of Black and White women in SWAN. Black women were more likely than their White counterparts to report socioeconomic disadvantage during their childhood (40.7% vs*.* 14.6%, respectively), characterized by a greater prevalence of low parental education (≤ high school), not owning a car or home, having received public assistance, and having had difficulties paying for food or rent [32]. Current family structures also differed by race: Black women were less likely than White women to be currently married (47.3% vs. 71.5%), more likely to have children (91.0% vs. 75.6%), and more likely to have more than two children (44.3% vs. 26.9%) [34]. Compared to White women, Black women recounted experiencing more adverse life events at study baseline and during the course of follow-up [35]. Specifically, Black women were significantly more likely to report events reflective of social and economic disadvantage, including financial stressors, new caregiving responsibilities, legal problems, trouble with the police, experiences of violence, as well as illness or death of close family members.

Black-White differences were also present in the lifestyles and behaviors ascertained in SWAN—cigarette use, alcohol consumption, and physical activity. Black women were more likely than White women to be current smokers (24.1% vs. 16.6%) and to be exposed to passive smoke for at least five person-hours weekly (39.4% vs. 29.1%). Conversely, however, a higher proportion of Black women were alcohol abstinent (56.9% vs. 39.1%). Black women also engaged in less leisure time exercise than White women [25, 34, 36],

### Direct Measures of the Experience of Discrimination

Black participants reported more discrimination than did White women on the Everyday Discrimination Scale. Black women had slightly higher scores on each of the individual items, and higher mean scale scores than White women (1.9 vs. 1.7, based on scale of 1 to 4, *p* < 0.0001) [37]. Notably, among Black women, the most commonly reported main reason was “race/ethnicity” (57%), whereas for White women the most commonly reported reason was “gender” (23%) with just 5% reporting “race” [38]. Women’s responses to individual items also differed by race, with ‘‘receiving poorer service in restaurants or stores’’ and ‘‘being treated as if you are dishonest’’ more likely to be endorsed by Black women than White women [37]. In a longitudinal latent class analyses, Black women were more likely than were White women to be classified in the group (Class 1) that had accumulated the highest perceived interpersonal discrimination over time, response domains, and attributed reason for discrimination [39].

### Age at Which the Final Menstrual Period Occurs

A central aim of SWAN was to describe the natural history of the menopause and the effects of this transition on the health of women. Age at the time of the final menstrual period (FMP) is the primary marker of ovarian senescence, signaling that ovulation is no longer possible. SWAN assessed factors associated with age at FMP in the screening survey and longitudinally in the cohort. In longitudinal analysis spanning 11 years of follow-up, Black women had a statistically significant 8.5 months earlier median age at menopause than did White women (52.17 years vs 52.88 years, respectively). Multiple adjustment for self-reported health, cigarette use, alcohol consumption, educational attainment, employment, past oral contraceptive use, weight, and physical activity reduced the Black-White disparity in age at menopause to 3.1 months, with the difference no longer statistically significant [34]. Importantly, as reviewed above, compared to White participants, Black participants have less education, greater cigarette use, and more unemployment—each of which are known to be influenced by structural racism [12, 40–44] and were all associated with an earlier age at FMP [34, 45].

The occurrence of ovarian and uterine surgery prior to natural menopause is an impediment to determining the age at which FMP occurs; germane to this review, Black-White differences exist in the experience of these surgeries [46, 47]. In the cross-sectional survey Black women were twice as likely as White women to have undergone hysterectomy/oophorectomy (30% vs 15%) [48], with Black women’s elevated risk continuing throughout cohort follow-up [49]. With surgical menopause, when both ovaries are removed, and simple hysterectomy, when menses are no longer manifest; age at FMP becomes clinically unobservable. In SWAN publications when computing natural age at FMP, women who underwent surgical menopause or simple hysterectomy were censored at the time of their surgery [34]. Thus, Black women were less likely to have the opportunity to experience a natural menopause and estimates of age at FMP in Black women are correspondingly influenced.

### Vasomotor Symptoms

Vasomotor symptoms (VMS), which include hot flashes and night sweats, are a cardinal symptom of the MT with 4 out of 5 women ever experiencing VMS [5, 50]; a central aim of SWAN was to elucidate women’s experience of these and other symptoms that occur as women transition from the reproductive to non-reproductive life stages. Corroborating and extending the work of prior cohort studies of the menopausal transition, longitudinal SWAN analyses of monthly calendars found that 5 to 8 years before the FMP about 20% of women report having hot flashes each month [51]. The monthly prevalence rises ~ 4 years before the FMP, reaching ~ 48% of women in the year prior to the FMP and then rises sharply at the time of the FMP to 60% of women. A year after the FMP the monthly prevalence begins to decline slowly.

Although this overall pattern of VMS timing applies to all racial/ethnic groups in SWAN, both in cross-sectional and longitudinal analyses of annual interview data Black women’s VMS burden exceeded that of White women, [50, 52–54]. At baseline, when women were pre- or early perimenopausal 46% of Black women compared to 37% of White women reported experiencing any VMS in the prior two weeks. After multivariable adjustment for other factors related to VMS (e.g., body mass index (BMI), smoking), Black women were 50% more likely than White women to have VMS [53]. In multiply adjusted, longitudinal analyses, the incidence of VMS was greater in Black vs. White participants in every stage of the MT [50, 51]. Frequent (> 6 days/week) and bothersome (self- assessed) VMS were each 60% more likely to occur in Black women than White women [55]. After 13 years of longitudinal follow-up that spanned the pre-to post-menopausal period, among all racial/ethnic groups VMS lasted for on average, at least7 years, continuing for ~ 4.5 years after menopause: Black women experienced VMS on average 10 years compared to 6.5 years for White women [56]. In a longitudinal trajectory analysis, Black women had threefold greater odds of consistently reporting any VMS from pre-menopause through post-menopause than did White women [57]. Lower educational attainment and greater perceived stress, more common in Black women, were related to longer duration of VMS. Despite their greater symptom burden, Black women were about half as likely as White women to use HT [34, 58].

### Depressive Symptoms and Major Depressive Disorders

At cohort entry, 27.4% of Black women compared to 22.3% of White women reported clinically significant depressive symptoms, on the CES-D [59]. A statistically significant Black-White difference in the presence of depressive symptoms did not persist after adjustment for factors that are related to depressive symptoms and more prevalent among Black women: lower education level, greater economic strain, poor health, lesser social support, greater perceived stress, and more stressful life events [59]. Subsequently race-stratified analyses were conducted to assess how risk profiles differed by race. Increased risk of elevated depressive symptoms was associated with self-reported poor health in Black women only; while lower education, presence of VMS, and anemia were associated with elevated depressive symptoms in White women only; and low social support, perceived stress, and experience of stressful events were associated with depressive symptoms in both groups [59]. Although adult socioeconomic disadvantage was not directly associated with depressive symptoms for Black women, in the Childhood Context study, low childhood SES and specifically childhood financial circumstances were associated with greater cumulative depressive burden (estimated by calculating area under the curve over 15 years of follow-up) for both Black and White women [32]. In the latent class analysis of discrimination, the group experiencing the highest perceived interpersonal discrimination (Class 1) had the highest risk of having an elevated CES-D score (> = 16) regardless of race/ethnicity; however as noted above, Black women were more likely than White women to be in Class 1 [39].

In the SWAN Mental Health ancillary study, Black women were more likely than White women to experience incident depression over 7 years of follow-up (20% vs. 13%) [6]. However, Black and White women did not differ in lifetime history of major depressive disorders, severity of depressive symptoms, or in rates of persistent/recurrent depression over 11 years of follow up [60] but Black women were more likely than White women to have numerous (> 7) recurrent episodes of depression over time. In race-stratified analyses**,** poorer role functioning and higher levels of depressive symptoms at baseline were associated with increased risk for persistent depression in Black women while sleep problems, low energy, and low optimism were associated with increased risk in White women. Predictors of a first episode of major depression were a lifetime history of an anxiety disorder, role limitations due to physical health, and a very stressful event. Notably, Black, compared to White, women were much less likely to have been treated for depression [6]. Reported treatment for emotional problems was 43% vs. 65%, treatment with psychotherapy was 20% vs. 36% and use of psychotherapeutic medications was 25% vs 36%, respectively.

### Sleep Disturbances

Black-White differences in subjective and objective sleep quality assessments were dissimilar with Black women less likely to self-report sleep problems but more likely to have objectively measured poor sleep quality. Overall in SWAN, self-reported difficulty falling asleep and staying asleep increased with advancing MT stage while early morning awakening decreased [61]. In adjusted longitudinal analyses, Black-White differences were not observed except that White women were more likely to report frequent awakenings than Black women. However, specific risk factors associated with Black women’s experience, specifically frequent VMS and less use of HT, were strongly related to sleep quality: those with more frequent VMS were twice as likely to report each measure of disturbed sleep, while post-menopause HT use halved the risk of difficulty falling asleep [34, 58].

Actigraphy-measures described a more overt picture of poorer sleep in Black vs. White women: average sleep duration was a half hour shorter in Black women, twice as many Black women slept < 6 h (40% vs. 19%), and Black women had more interrupted sleep, averaging about 10 more minutes of awake time after sleep onset [62]. Black-White disparities in actigraphic measures of sleep quality persisted after adjustment for age, use of sleep medications, and measures of social disadvantage including education and employment and were mediated by financial hardship, number of health problems and stressful life events, physical inactivity and frequency of VMS.

In the ancillary Sleep Study, sleep polysomnography averaged across two nights had results concordant with the actigraphy data described above: Black women had worse sleep efficiency (a ratio of time asleep divided by time in bed), took longer to fall asleep, and spent more time awake after sleep onset. Slow-wave sleep, the deepest wave of NREM sleep, was lower while cortical arousal was higher in Black women [62]. In adjusted analyses, both race and financial strain were associated with less sleep efficiency. In additional adjusted analyses, everyday discrimination was associated with lower self-reported sleep quality and with increased polysomnography-assessed wake after sleep onset, a measure of sleep continuity [63]. In the full study sample, sleep disordered breathing was associated with worse inflammatory profiles – greater levels of C-reactive protein (CRP), fibrinogen, and plasminogen activator inhibitor 1 (PAI-1). In Black women only, shorter sleep duration was associated with higher CRP levels and lower sleep efficiency and greater numbers of awakenings after sleep onset were associated with higher fibrinogen levels [64].

### Physical Health: Burden at Enrollment

At baseline, when women were premenopausal or beginning the MT, Black women already had a greater disease burden than White women. Not only were they more likely than White women to self-report being in poor health (16.2% vs. 6.6%), they were also more likely to be obese (51.3% vs. 31.0%) [34] and to have diabetes (10.7% vs. 4.0%), metabolic syndrome (MetS) (26.2% vs. 18.4%), and hypertension (36.9% vs. 16.2%) [65]. Black women also had an increased risk of cardio-metabolic disease onset over time (see below). In addition, allostatic load, a cluster measure of physiologic dysregulation across multiple systems (cardiovascular, metabolic, inflammatory, and neuroendocrine markers [66]) was higher at baseline in Black women than White women (3.7 vs. 2.3, reflecting the number of markers with levels in their respective highest risk quartile). Notably, both the allostatic load at baseline and increase over 8 years was mediated through discrimination, perceived stress, and hostility [67]. Furthermore, an assessment of OA at the Michigan site documented that Black women were more likely to have radiographic evidence of knee (23.2% vs. 8.5%) and hand OA (25.5% vs. 17.4%) than White women with the positive predictive value of knee pain significantly higher in Black women compared to White women Table 3.

**Table 3:** Health Burden of Black and White Women at Cohort Enrollment: Study of Women’s Health Across the Nation, 1996–1997

	Black Women	White Women
Poor self-reported health^a^	16.2%	6.6%
Obese (> = 30 kg/m^2^)^a^	51.3%	31.0%
Diabetes Mellitus^b^	10.7%	4.0%
Metabolic Syndrome^b^	26.2%	18.4%
Hypertension^b^	36.9%	16.2%
Allostatic Load (mean)^c^	3.7	2.3

^a^*n* = 916 Black women, 1533 White women [34]; ^b^*n* = 875 Black women, 1496 White women; Metabolic Syndrome defined as meeting at least 3 of the 5 criteria: triglycerides >  = 150 mg/dL; HDL cholesterol < 50 mg/dL; systolic blood pressure >  = 130 mmHg, diastolic blood pressure >  = 85 mmHg, or on antihypertensive medication; fasting glucose > 10 mg/dL and/or diabetes; and waist circumference >  = 88 cm [65]; ^c^*n* = 796 Black women, 1399 White women; Allostatic Load defined as the number of 11 biomarkers in the highest risk quartile (systolic and diastolic blood pressure, total cholesterol, high-density lipoprotein cholesterol, triglycerides, body mass index, waist-to-hip ratio, fasting serum glucose, C-reactive protein, fibrinogen, Dehydroepiandrosterone sulfate) [67]

### Physical Health: Metabolic Syndrome (MetS) and Diabetes

At enrollment, 21% of SWAN participants had MetS, defined as having at least 3 of the following: obesity, high triglycerides, low high-density cholesterol, impaired fasting glucose, or hypertension. An additional 12.8% developed MetS by the fifth follow-up visit [68]. As noted above, Black women were more likely than White women to have MetS at enrollment. They also had a 25% increased odds of developing MetS during follow-up. Similarly, Black women had more diabetes at baseline [65], and also had a higher incidence of diabetes (5.9% vs. 2.7% for White women) over the first 3 years of follow-up [69].

Depression was an important risk factor for incident diabetes and a significant interaction existed between depression and race. Depression was associated with a 2.5-fold increase in the odds of incident diabetes in Black women (95% CI = 1.27–5.15) but not in White women, suggesting that Black women may be differentially vulnerable to the impacts of depression on metabolic disease. When SWAN modeled symptom burden by examining latent symptom clusters, a high symptom burden (e.g., reporting multiple severe physical, psychological, and menopausal symptoms) at baseline was associated with both baseline prevalence and earlier onset of diabetes and MetS compared to women with few and minor symptoms [70]. After adjustment for baseline symptom burden and socioeconomic and lifestyle risk factors, onset of diabetes was 24% earlier in Black women (95% CI 17.2–30.8% earlier) compared to White women, a finding partially mediated by waist circumference. Similarly, onset of MetS was 15.3% (10.0, 20.3) earlier in Black women compared to White women [70, 71].

Peripheral neuropathy is a significant consequence of diabetes [72], but SWAN has documented a high prevalence of peripheral neuropathy in midlife women without diabetes [73, 74]. Although, the prevalence of peripheral neuropathy did not differ between Black and White women, prevalence was associated with health inequities (higher BMI, and diabetes), social disadvantage (financial strain, smoking) and everyday discrimination. Each unit increase in discrimination was associated with 29% higher odds of having peripheral neuropathy independent of other risk factors with a ~ 30% of the total effect of discrimination mediated through BMI [74].

### Physical Health: Cardiovascular disease (CVD)

At baseline, Black women had a higher 10-year risk of myocardial infarction or coronary death with a higher mean Framingham risk score and a higher mean composite burden score than White women [75], although differences were attenuated after adjustment for education. The percentage with risk scores > 20 (signifying an 11% increase in 10-year risk) were 2.1% and 0.9%, respectively. In women without a history of stroke, who were not using antihypertensive or antidiabetic medications at baseline, higher everyday discrimination was associated with increasing systolic (beta = 2.19, 95% CI: 1.21, 3.16) and diastolic (beta = 0.99; 95% CI: 0.41, 1.56) blood pressure during follow-up [76], associations that persisted even after multivariable adjustment and was mediated through adiposity.

#### Subclinical CVD

Data from SWAN Heart suggest that patterns of and risk factors for subclinical CVD also differ by race/ethnicity. Carotid plaque was significantly associated with a fourfold higher risk of aortic calcification (AC) in White women; however, in Black women plaque was associated with a 25% reduction in risk of AC, suggesting that plaque may not be an appropriate marker of calcification in Black women possibly due to differences in calcium metabolism [77]. In the full SWAN cohort, Black women were found to have significantly less plaque but significantly greater common carotid artery intima-media thickness (IMT) [78]. Differences in risk profiles were also observed for pulse wave velocity (PWV), a measure of arterial stiffness. PWV was associated with increasing waist circumference and systolic blood pressure; however, the slope for systolic blood pressure was steeper in Black women compared to White women. In addition, higher low-density lipoprotein cholesterol (LDL-C), diastolic blood pressure, and glucose levels were associated with progression only in Black women [79], and associations with CRP were also observed to be stronger in Black women [80].

Psychosocial risk factors for subclinical CVD also differed between Black and White women. Multiple upsetting losses across 12 years of follow-up were associated with IMT among Black women but not White women [81]. In SWAN Heart, depressive symptoms were associated with higher overall AC (*p* < 0.0001) and with increased prevalence of high AC >  = 100 (*p* = 0.001) in Black, but not White women [81]. Additionally, cumulative stress burden and everyday discrimination were associated with higher average IMT in Black but not White women [82]. Within Black women, when everyday discrimination was averaged across 5 visits to assess chronic discrimination, chronic discrimination, but not concurrent discrimination, was significantly associated with CAC [83], associations not observed among White women (unpublished).

In contrast to the above findings, in an analysis conducted after more than 10 years of follow up among women without CVD in the full cohort, chronic everyday discrimination was only associated with IMT in White women [84], despite White women’s consistently lower reports of experiencing discrimination. Potential reasons for this discrepant finding include differential loss to follow up, and earlier development of CVD among Black, compared to White, women in SWAN, which may have removed the most vulnerable Black women from this analysis attenuating potential associations. It is also possible that Black women are most vulnerable to the effects of discrimination earlier in midlife, as some research has shown that reports of discrimination among Black adults are relatively high in young adulthood and midlife, but much lower in older age [85, 86].

#### Inflammation

is an independent risk factor for cardiometabolic disease [87]. Black women had higher median CRP concentrations (3.2 mg/L) than White women (1.5 mg/L) and they were approximately 40% more likely to have a CRP greater than 3 mg/L. [65]. Notably, CRP is strongly correlated with BMI (0.63 *p* < 0.0001). When the association between inflammation and everyday discrimination was examined, everyday discrimination was associated with higher CRP levels only in women who were not obese (BMI < 30) [88]. Adipokine levels also differed by race. Compared to White women, Black women had lower levels of the cardiovascular protective adipokines, total adiponectin (3.40 μg/mL lower) and high molecular weight adiponectin (beta =  − 0.53 μg/mL lower), but higher levels of leptin (3.26 μg/mL) after adjustment for fat mass and other covariates [89].

#### Medical Treatment

At baseline, peri-menopausal Black women were more likely than White women to meet criteria for recommending preventive intervention based on the Joint National Committee on Prevention, Detection, Evaluation, and Treatment of High Blood Pressure (JNC 7) and the National Cholesterol Education Program’s Adult Treatment Panel (ATP III) screening guidelines [90]. However, they were more likely to have untreated hypertension (16.9% vs. 7.9%) and, given treatment, less likely to have controlled hypertension (50.0% vs. 76.8%). At follow visit 12, we evaluated statin use [91]: 32% of Black women and 29% of White women met the 2013 American Heart Association (AHA) and American College of Cardiology (ACC) guidelines criteria for statin use. Among those who met the criteria, 41% of Black women and 49% of White women reported using statins. After adjustment for sociodemographic and clinical covariates, statin-eligible Black women had half the odds of using statins compared to White women. Notably, statin-eligible Black women (56%) were more likely to report difficulty paying for basics compared to White women (29%).

### Self-Reported Physical Function and Measured Physical Performance

In the large screening survey that SWAN conducted to identify eligible women and recruit them into the cohort, Black women were 22% more likely to report having substantial limitations in physical functioning relative to White women [92]. The SWAN cohort first measured self-reported physical function at the 4^th^ follow up and then continued to inquire about it longitudinally. The Black-White physical function disparity persisted in longitudinal follow up: By the 12^th^ follow-up visit, Black women’s odds of having substantial functional limitations were 63% higher than those of White participants, with just 71% of Black women reporting no limitations compared to 80% of White women [73].

Michigan and Chicago, two SWAN sites that enrolled Black and White women, collected longitudinal, objective measures of physical performance. At the outset, Black women’s stair climb times were 5% slower; this offset between Black and White women persisted over time, although rates of decline (slopes) were similar [93]. Measured physical performance was added to the full SWAN cohort protocol at the 13^th^ follow-up and the overall mean score, derived from a summed decile ranking of each of 3 assessments (grip strength, timed 4-m walk, and timed chair stands), was 19% percent higher, with higher scores meaning worse function, in White compared to the Black women [94]. The Black disparity in overall physical performance score was explained largely (76%) by mediators related to social disadvantage, specifically, lower educational level, presence of financial strain, lower levels of self-reported physical activity and higher BMI.

Chronic medical conditions negatively influence physical function and performance; Black-White disparities in the prevalence and incidence of major diseases account for some of the observed disparity in physical performance and physical disability [95–98]. In the full SWAN sample, each additional chronic medical condition present at baseline was associated with a 4.0% poorer self-reported baseline physical functioning score; each additional baseline medical condition also increased the score’s annual rate of decline by 0.20% yearly. Development of an incident chronic medical condition was associated with an additional 1.9% poorer score and a 0.33% yearly rate of decline per condition [96]. As noted above, Black were women more likely than White women to start the study with prevalent, and then to develop, incident hypertension, diabetes mellitus, MetS, and OA [97]. Additionally, at the 12^th^ follow-up visit the Michigan site administered the World Health Organization Disability Assessment Schedule (WHO-DAS): Black women were twice as likely as White women to report moderate to extreme global disability [98].

### Health-related quality of life (HRQL)

As noted above, SWAN initially assessed 5 HRQL domains – vitality, bodily pain, social function, role limitations due to physical health, and role limitations due to emotional health [29]. Black women began SWAN with less favorable HRQL than did White women: ~ 30% vs. ~ 20% scored in the worst quartiles of social function and pain, respectively [29]. The Black-White disparity in social function remained statistically significant after adjustment for characteristics reflecting social disadvantage including educational level and financial strain, and risk factors more common in Black women including arthritis, poor sleep and VMS, lifestyle factors (smoking and physical activity), BMI, and stress (perceived stress and upsetting life events). Statistically significant racial disparities in pain also remained after adjustment for indicators of social disadvantage and health but diminished when adjusted for lifestyle factors [9]. A subsequent analysis of the pain domain considered everyday discrimination and found discrimination to be positively associated with pain in in both Black and White women. The magnitude of the association was stronger in White women, but Black women reported more discrimination [99].

SWAN ascertained the complete SF-36 beginning at the 6^th^ follow-up, permitting computation of the standard summary scores – the physical health component (PCS) and mental health component (MCS) (see methods) [100]. Over 11 years of follow-up, the PCS declined by 4.5 points and the MCS increased by 3.9 points (each scale ranges from 1–100 points). Black women’s unadjusted PCS trajectory over time was 3.6 points lower than White women, while the trajectory for MCS did not differ. Multivariable analyses adjusting for factors associated with social disadvantage including financial strain, number of medical conditions, sleep problems, smoking, and less leisure physical activity obviated the Black-White disparity in PCS. Again, because these risk factors are more common in Black women, adjusting for them may obscure an important pathway underlying this observed disparity.

## Discussion

This paper summarizes evidence of disparities in reproductive aging and midlife health between US Black and White women in SWAN, emphasizing the role of social disadvantage, which we argue is likely related in part to structural racism, as an important driver of many of these disparities. In SWAN, Black women entering the midlife were more likely to report life contexts that increase the risk for having earlier menopause, more vasomotor symptoms, and greater chronic disease burden compared to White women. On average, principal differences in Black-White life contexts included a lower educational level, more financial strain, a lower likelihood of being married or being employed, greater cigarette use, being less physically active, experiencing more upsetting life events and reporting more discrimination among Black women. Black women were also less likely to report treatment for VMS, depression, and hypertension.

Black-White disparities in timing of reproductive aging and the burden of VMS depression, and poor sleep during the menopausal transition were also documented. On average, Black women had their final menstrual period more than 8 months earlier than White women. This disparity in age at menopause is clinically relevant: earlier age at menopause is associated with increased risks of CVD and mortality [101]. With respect to symptom burden, Black women experienced more frequent, severe, and persistent VMS, were more likely to have new onset and more episodes of depression, and had poorer objectively assessed sleep quality than did White women. Moreover, Black women were less likely to receive treatment for hot flashes and depression. Disparities in VMS are important not only due to the discomfort and bother they cause, but also because VMS are associated with more sleep disturbances [7, 61, 102] and greater cardiovascular risk [103, 104]. With respect to depressive symptoms and major depressive disorder, Black women had higher rates of depressive symptoms and in some instances, higher rates of incident depression compared to White women. The risk of depression during midlife was elevated by the presence of both childhood and adult financial strain, each more common in Black than in White women. This is important because apart from its contribution to poor mental health, depression is also a risk factor for diabetes and heart disease [105, 106], outcomes known to be more prevalent in Black compared to White women. Interestingly, SWAN observed an impact of depression on diabetes risk only in Black women. Objective measures of sleep quality revealed important Black-White disparities that were not apparent by subjective report. Poor sleep is emerging as an important determinant of cardio-metabolic risk [64, 107] and cognitive function [108, 109]. Observed associations between poor sleep quality with CRP, PAI-1 and fibrinogen suggest that inflammation and pro-coagulation processes may underlie relationships between poor sleep and cardio-metabolic risk; moreover, these mechanistic pathways appear to differ by race, with inflammatory pathways being particularly important for Black women [64].

Black-White disparities in health across the midlife were also documented. Black women entered the menopausal transition with a more adverse cardio-metabolic health burden including a higher prevalence of obesity, MetS, diabetes, and hypertension, and osteoarthritis; and, subsequently, had a higher incidence and earlier onset of cardio-metabolic disease than White women. Disparities in treatment were documented for hypertension. Taken together, SWAN findings suggest that throughout the early stages of midlife, compared to White women, Black women may be at a differential risk of atherosclerotic disease due to the impact of depressive symptoms, cumulative stress, midlife loss, and chronic discrimination. VMS and arterial stiffening may be an additional pathway contributing to Black women’s increased risk of CVD [110]. Black women also had earlier onset of OA, worse physical functioning, more disability and reduced HRQL, specifically in the areas of pain and social functioning, than White women. Midlife is a critical period for onset of physical functioning decline, which significantly worsens quality of life [111, 112]. Declines in physical functioning are risk factors for increasing disability and greater chronic disease [111].

In some instances in SWAN, racial health disparities could be accounted for by Black-White differences in risk factors that are associated with social disadvantage, and, in other cases, disparities were mediated by these factors. However, higher rates of VMS, poorer sleep quality, greater bodily pain and higher CVD risk in Black women remained after multiple adjustment including for socio-economic status and available measures of social disadvantage in SWAN, suggesting that other, non-measured factors (including unmeasured factors potentially related to structural racism), may also help explain these disparities. Differences in the life contexts of Black and White SWAN participants correspond to the social disadvantage experienced by Black women in the general US population. For example, the gap observed in SWAN between educational attainment and economic resources among Black women potentially reflects structural factors related to diminished opportunities for Black women, such as the ongoing Black-White wage gap and/or the lower return on education for Black versus White adults [40–42]. This suggests that the traditional approach of simply adjusting for the range of factors that characterize women’s life context may actually obscure scientific understanding of how the magnitude of racial health disparities and Black women’s midlife health may be shaped by structural racism.

Black-White differences in health outcomes may also be related to more unfavorable behavioral profiles in Black women—also potentially driven in part by structural racism. Greater cigarette use in Black Americans is consistent with disproportionate advertising and access to tobacco products in Black communities [43, 44]. Lower levels of leisure time physical activity in Black women may be attributable to the higher likelihood of Black Americans living in less safe communities with reduced access to green spaces, as well as having less free time to exercise secondary to financial constraints [113]. Although epidemiologic analyses frequently aim to identify the independent association of race/ethnicity with health outcomes after adjusting for such factors, this practice obscures the fact that the distribution and clustering of socioeconomic, lifestyle and health characteristics differ across race, are structurally determined [12], and are important pathways through which structural racism contributes to health disparities.

The impact of everyday discrimination has not been systematically examined across the range of health outcomes assessed in SWAN. However, when evaluated, discrimination was linked to each outcome examined including worse sleep, higher levels of pain, higher blood pressure, higher prevalence of coronary calcium, greater IMT, higher probability of having peripheral neuropathy, and higher CRP levels. Black women in SWAN consistently reported experiencing more everyday discrimination than did White women and were more likely to endorse ‘‘receiving poorer service in restaurants or stores’’ and ‘‘being treated as if you are dishonest’’, consistent with the “Shopping While Black” phenomenon—i.e., consumer racial profiling—which has been found to more frequently impact Black, compared to White, consumers [37, 114–116]. The impact of discrimination did not differ by race for sleep, blood pressure, or peripheral neuropathy and was stronger in White women than Black women for pain; however, for subclinical CVD an association existed for Black women only. Furthermore, not only did Black women report more discrimination but also, for many outcomes, relationships with discrimination were mediated by adiposity. Given that Black women had a higher prevalence of obesity at baseline, it is plausible that Black women entered SWAN already expressing the health impact of discrimination.

We acknowledge several limitations of SWAN in the context of studying racial disparities in health. First, as a study of the natural menopause transition, the cohort necessarily excluded women who had previously undergone a hysterectomy, bilateral oophorectomy, or had achieved a natural menopause. Black women were, therefore, less likely to be eligible for the cohort than were White women, primarily because of large differences in the prevalence of having had a hysterectomy/bilateral oophorectomy (30.2% vs. 15.4%, respectively) [48]. As surgical menopause is a risk factor for cardiovascular morbidity and earlier mortality [117–119], Black participants in SWAN may represent a relatively healthier subset of their source population compared to White participants in these respects. Second, the sample of Black women in SWAN was recruited from industrial, urban areas of the Midwest and Northeast, with White women sampled from these same regions and from a city in New Jersey and two urban areas in California. Therefore, SWAN’s findings may not be generalizable to rural women or women from other US regions, including the South. Finally, since its inception, SWAN included a measure of interpersonal discrimination, but it did not directly assess structural racism. Thus, in this review, we explicitly considered the socio-historical context in which participants were born, came of age, and sought access to health care prior to midlife and conceptualized measures of social disadvantage as potential proxy measures of the structural racism that has permeated the lives of Black women of this generation.

SWAN strengths include recruiting participants using community-based random sampling methods, including women of five races/ethnicities, and retaining 75% of the surviving cohort for over 25 years. Each of these attributes enhances the generalizability and representativeness of study results. SWAN prospectively assessed comprehensive measures of the menopause transition (e.g., timing, endogenous hormone trajectories, and symptoms) and concurrent, repeated measures of physical and psychological health. Observations spanned participants’ 5^th^ to 7^th^ decades of life (ages 40–69 years). Importantly, SWAN collected data on proxies of structural racism (e.g., education, employment, income) and utilized validated scales to measure quality of life and everyday discrimination. This multi-faceted data permits identification of complex inter-relations among health and well-being, social disadvantage (e.g., education, employment opportunity, ability to pay for basics such as food and rent), adverse life events (e.g. legal problems or experience of violence) and the experience of discrimination.

Despite SWAN’s considerable strengths, it should be noted that SWAN was not explicitly designed to evaluate the impact of structural racism on health. Although one manifestation of racial disparities is the earlier onset of disease and disability, to date, SWAN has not generally employed analytical strategies that account for differential baseline prevalence when estimating disease burden overtime. Publications that focused on continuous outcomes, such as physical function and inflammatory markers, more commonly considered racial differences in both baseline levels and trends over time, which have yielded important information regarding the timing of risk stratification. Although somewhat ahead of its time in incorporating a validated measure of discrimination, this measure has not been systematically included as an explanatory variable in most SWAN analyses, thus our knowledge of its impact on midlife health, including risk of VMS, remains circumscribed. Key variables such as health insurance status were not measured consistently across visits.

## Conclusion

SWAN was designed more than 25 years ago to study Black, Chinese, Japanese, Hispanic, and White women in the US as they transitioned from pre- to post-menopause. The study has advanced our understanding of the MT significantly and has identified numerous midlife health factors that contribute to successful aging. Although SWAN did not explicitly assess structural racism, in contrast to other epidemiologic cohorts of the same era, SWAN did assess multiple measures of social disadvantage, stressors that reflect inequities stemming from structural factors, and included a measure of discrimination. As summarized in this review, SWAN has documented that Black women’s experience of reproductive aging differs from that of White women and that Black women enter midlife with a more adverse cardio-metabolic profile and more physical limitations. Consequently, early interventions on blood pressure, LDL, and waist circumference in midlife may be key to reducing Black women’s CVD risk, as would reductions in the disparities in treatment for hypertension. Increased attention to the role of structural factors and discrimination on midlife health is also warranted.

### Recommendations

Other publications have provided recommendations regarding the measurement of structural racism and methods for assessing health disparities [120, 121]. Reflecting on SWAN’s findings, we offer the following recommendations for enhancing epidemiologic investigations of racial health disparities potentially driven by structural racism in cohort studies.

- Consider how selection may differ by race when constructing a cohort. Design decisions should aim to minimize differential selection bias, with enhanced attention to issues of left truncation and left censoring.
- Publish baseline distributions of study variables by race/ethnicity to explicit documentation and consideration of sample differences, particularly in prevalence of social disadvantage and age of disease onset, their potential causes, and their implications for disease risk at enrollment and across time.
- Incorporate measures of discrimination and systematically assess the impact of exposure to discrimination (analogous to current practice with smoking exposures).
- Give more priority to examining structural causes of racial difference: Systematically evaluate the ‘macrolevel systems, social forces, institutions, ideologies, processes’ [23] and policies that cause the clustering of adverse demographic, socioeconomic, and environmental factors by race.

## Data Availability

Public use data sets are available at https://www.icpsr.umich.edu/icpsrweb/ICPSR/series/253 and at https://www.nia.nih.gov/research/dgcg/study-womens-health-across-nation-swan-repository

## Abbreviations

AC: Aortic calcification
BMI: Body mass index
CAC: Coronary artery calcification
CES-D: Center for Epidemiologic Studies Depression scale
CRP: C-reactive protein
CVD: Cardiovascular disease
HRQL: Health-related quality of life
HT: Hormone therapy
IMT: Intima-media thickness
LDL -C: Low-density lipoprotein cholesterol
MCS: Mental health component summary score
MetS: Metabolic syndrome
MT: Menopausal transition
PCS: Physical health component summary score
SES: Socioeconomic status
SF-36: RAND 36-item Short Form Survey
SWAN: The Study of Women’s Health Across the Nation
US: United States
VMS: Vasomotor symptoms

## Footnotes

1 In the century following the abolition of slavery (with the ratification of the Thirteenth Amendment in 1865), a collection of local and state legal codes, together known as Jim Crow laws, were passed to enforce the legal segregation and discrimination of individuals on the basis of race [20]. Many of these laws remained in place until the passage of the 1964 US Civil Rights Act, which prohibits “discrimination or segregation on the basis of race, color, religion, or national origin” [22]. Commonly associated with the American South, legal and de facto segregation and discrimination of Black Americans extended to localities throughout the country, and its legacy has persisted beyond the passage of the Civil Rights Act of 1964. [21]
