# Correction: Disparities in Reproductive Aging and Midlife Health between Black and White women: The Study of Women’s Health Across the Nation (SWAN)

**DOI:** 10.1186/s40695-022-00082-x

**Published:** 2022-10-21

**Authors:** Siobán D. Harlow, Sherri-Ann M. Burnett-Bowie, Gail A. Greendale, Nancy E. Avis, Alexis N. Reeves, Thomas R. Richards, Tené T. Lewis

**Affiliations:** 1 Department of Epidemiology, University of Michigan, School of Public Health, United States, 1415 Washington Heights, Ann Arbor, MI 48104-2029 USA; 2 Endocrine Division, Department of Medicine, Massachusetts General Hospital, Harvard Medical School, Boston, USA; 3 Division of Geriatrics, David Gefen School of Medicine, University of California at Los Angeles, Los Angeles, USA; 4 Department of Social Sciences & Health Policy Wake Forest School of Medicine, Winston-Salem, USA; 5 Department of Epidemiology, School of Public Health, University of Michigan, Ann Arbor, USA; 6 Department of Epidemiology, Rollins School of Public Health, Emory University, Atlanta, USA

**Correction: Womens Midlife Health 8, 3 (2022)**

**https://doi.org/10.1186/s40695-022-00073-y**

Following publication of the original article [1], we have been notified that the article text had a mistake. The correct text is should be as per below:

Michigan and Chicago, two SWAN sites that enrolled Black and White women, collected longitudinal, objective measures of physical performance. At the outset, Black women’s stair climb times were 5% slower; this offset between Black and White women persisted over time, although rates of decline (slopes) were similar [93]. Measured physical performance was added to the full SWAN cohort protocol at the 13th follow-up and the overall mean score, derived from a summed decile ranking of each of 3 assessments (grip strength, timed 4-m walk, and timed chair stands), was 19% percent higher, with higher scores meaning better function, in White compared to the Black women [94]. The Black disparity in overall physical performance score was explained largely (76%) by mediators related to social disadvantage, specifically, lower educational level, presence of financial strain, lower levels of self-reported physical activity and higher BMI.

## References

[1] Harlow SD (2022). Disparities in Reproductive Aging and Midlife Health between Black and White women: The Study of Women’s Health Across the Nation (SWAN). Womens Midlife Health.

